# Self-face and emotional faces—are they alike?

**DOI:** 10.1093/scan/nsab020

**Published:** 2021-02-08

**Authors:** Anna Żochowska, Maria M Nowicka, Michał J Wójcik, Anna Nowicka

**Affiliations:** Laboratory of Language Neurobiology, Nencki Institute of Experimental Biology, Polish Academy of Sciences,voivodeship mazowieckie,Warsaw 02-093, Poland; Laboratory of Language Neurobiology, Nencki Institute of Experimental Biology, Polish Academy of Sciences,voivodeship mazowieckie,Warsaw 02-093, Poland; Department of Experimental Psychology, University of Oxford,Oxfordshire, Oxford OX2 6GG,UK; Laboratory of Language Neurobiology, Nencki Institute of Experimental Biology, Polish Academy of Sciences,voivodeship mazowieckie,Warsaw 02-093, Poland

**Keywords:** self, emotion, familiarity, ERP, RSA

## Abstract

The image of one’s own face is a particularly distinctive feature of the self. The self-face differs from other faces not only in respect of its familiarity but also in respect of its subjective emotional significance and saliency. The current study aimed at elucidating similarities/dissimilarities between processing of one’s own face and emotional faces: happy faces (based on the self-positive bias) and fearful faces (because of their high perceptual saliency, a feature shared with self-face). Electroencephalogram data were collected in the group of 30 participants who performed a simple detection task. Event-related potential analyses indicated significantly increased P3 and late positive potential amplitudes to the self-face in comparison to all other faces: fearful, happy and neutral. Permutation tests confirmed the differences between the self-face and all three types of other faces for numerous electrode sites and in broad time windows. Representational similarity analysis, in turn, revealed distinct processing of the self-face and did not provide any evidence in favour of similarities between the self-face and emotional (either negative or positive) faces. These findings strongly suggest that the self-face processing do not resemble those of emotional faces, thus implying that prioritized self-referential processing is driven by the subjective relevance of one’s own face.

## Introduction

The self-face—as a unique piece of self-referential information—is strongly linked to the physical self-identity (McNeill, 1998; Estudillo, 2017). Within the vast number of faces encountered during everyday life, there is perhaps no face that has more meaning to us than our own face. It has even been suggested that the image of one’s own face may trigger the sense of self-awareness in general (Keenan *et al.*, 2005; Devue and Brédart, 2008). A growing literature shows the prioritized processing of that stimulus and provides converging lines of evidence indicating that one’s own face captures attention in various conditions and on different levels of processing (for review see: Humphreys and Sui, 2016).

There is an ongoing discussion whether the prioritized processing of the self-face is a consequence of its high familiarity, resulting from frequent exposure to one’s own image in mirrors and on photographs (e.g. Bortolon *et al.*, 2018). Numerous studies have compared processing of the self-face to the processing of faces that are less familiar. For instance, behavioural studies have shown that when participants were asked to classify faces as belonging to themselves, a friend or a stranger, classification of the self-face was much faster than classification of the other’s faces (Keyes and Brady, 2010; Keyes, 2012). Moreover, a stronger interference was generated by a self-face flanking a classmate’s name in comparison to the reverse condition, i.e. a classmate’s face flanking a self-name (Brédart *et al.*, 2006). The self-face was also more quickly detected amongst distracters than a stranger’s face, even if it was presented in an atypical orientation and after hundreds of trials (Tong and Nakayama, 1999). Functional magnetic resonance imaging (fMRI) studies revealed increased activation of neural regions, such as the medial prefrontal cortex and anterior cingulate cortex, to images of one’s own face when compared with other’s faces (Keenan *et al.*, 2000; Kircher *et al.*, 2001; Heatherton *et al.*, 2006). Event-related potential (ERP) studies, in turn, showed that brain activity associated with self-face processing is enhanced compared to the processing of familiar, famous and unknown faces (Keyes and Brady, 2010; Miyakoshi *et al.*, 2010; Tacikowski and Nowicka, 2010; Tacikowski *et al.*, 2011; Cygan *et al.*, 2014; Kotlewska and Nowicka, 2015; Alzueta *et al.*, 2019). Those results may be viewed as evidence that the pre-experimental familiarity of processed faces determines a commonly reported pattern of findings: the strongest brain responses to the self-face (i.e. extremely familiar face) and the weakest to unknown faces, with familiar/famous faces in between (e.g. Tacikowski and Nowicka, 2010).

However, the notion of the extreme familiarity of the self-face as the driving factor of its special processing status is undermined by experiments revealing that even abstract stimuli arbitrarily associated with the self during the experiment benefit from a robust prioritization effect despite previously being both unfamiliar and self-irrelevant (e.g. Sui *et al.*, 2012, 2014). In a similar manner, an unfamiliar face that was also arbitrary associated with the self can be preferentially processed (Woźniak *et al.*, 2018). In that study, three unfamiliar faces were introduced with the labels ‘you’, ‘friend’ and ‘stranger.’ Afterwards, participants were required to assess whether two stimuli presented in succession (i.e. face and label) matched. If the first stimulus (either the ‘new’ face or the label) referred to the self, reaction times (RTs) were faster. The prioritized processing of initially unfamiliar stimuli—that do not have an intrinsic relation but an acquired relation to the self—seems to contradict the notion that familiarity is the driving factor of preferential self-referential processing. In addition, there is evidence that the self-face is preferentially processed even when compared with faces that share a similar level of familiarity; this includes a close-other’s face, e.g. mother’s, father’s, sibling’s, partner’s, etc. (Cygan *et al.*, 2014; Kotlewska and Nowicka, 2015; Kotlewska *et al.*, 2017), and the faces of dizygotic twins (Butler *et al.*, 2013).

Finally, in a recent meta-analysis study, RTs for the self-face were compared with RTs for other faces across a large number of studies (Bortolon and Raffard, 2018). The tested moderators included the familiarity (i.e. whether the face was familiar or not to the participants) and identity of faces (i.e. whether the face belonged to someone personally known by participants or whether it was a famous person or a stranger). The results of that study illustrate that RTs were substantially shorter in response to the self-face than to other faces in general. However, none of the two aforementioned moderators had an impact on this RT effect (Bortolon and Raffard, 2018). Altogether, the mentioned findings may suggest the involvement of factors other than familiarity in the preferential processing of the self-face.

It is worth noting that self-related stimuli differ from stimuli referring to other people not only in respect of their familiarity levels but also in respect of their subjective emotional relevance. Current definitions of emotions emphasize their subjective character (e.g. Dolan, 2002). Therefore, it is the personal relevance of a particular stimulus that determines its emotional *vs* neutral evaluation. In contrast to the familiarity factor, the role of emotional aspects in prioritized self-face processing has gained much less empirical attention. However, there is indirect evidence suggesting some substantial similarities in the processing of one’s own face and emotional faces. For instance, both types of faces capture, hold and bias attention (Eimer and Kiss, 2007; Wieser *et al.*, 2018; Wójcik *et al.*, 2018, 2019). In addition, both emotional faces and the self-face can be processed without awareness (Zotto and Pegna, 2015; Wójcik *et al.*, 2019). Those findings may suggest that the self-face, like emotional faces, can be treated as a salient stimulus.

The processing of both of these stimuli shares similar neuronal implementations (Northoff, 2016). Specifically, similar patterns of ERP findings were observed for the self-face compared to other faces and emotional faces compared to neutral faces. In both cases significantly enhanced amplitudes of late ERP components were typically reported, both to the self-face and emotional faces (e.g. Luo *et al.*, 2010; Tacikowski and Nowicka, 2010; Kotlewska and Nowicka, 2015). Moreover, the processing of any type of emotion, either positive or negative, was shown to activate the anterior cortical midline structures as well as the ventromedial and dorsomedial prefrontal cortex (e.g. Phan *et al.*, 2002; Etkin *et al.*, 2011; Roy *et al.*, 2012; Rolls, 2019), i.e. the very same regions recruited in various self-referential processes (e.g. Moran *et al.*, 2006; Northoff *et al.*, 2009), including self-recognition (Keenan *et al.*, 2000, 2001; Kircher *et al.*, 2001; Heatherton *et al.*, 2006). This overlap may indicate that exposure to self-face induces both introspection and emotional reactions effectively. In a similar vein, it was proposed (Devue and Brédart, 2011) that self-recognition preceded by the perception of one’s own face may cause a cascade of higher-order cognitive operations: information that is identified as related to oneself can be evaluated in terms of its relevance to current goals, expectations, etc. The result of such an evaluation may be accompanied by emotional responses (Craver, 2003; Morita *et al.*, 2008).

The goal of the present ERP study was to directly compare the neural correlates of self-face and emotional face processing. On the basis of the self-positivity bias (Greenwald, 1980; Watson *et al.*, 2007) and the theory of implicit positive association (IPA) with the self (Ma and Han, 2010), one may assume that self-face is treated and processed like an emotionally positive face (i.e. a happy/smiling face). The self-positivity bias is one of the most common findings in social psychology (Dunning *et al.*, 2004; Alicke et al., 2005). It has been found that people have a basic desire to feel good about themselves (James, 1890/1950) and possess a rather positive view of the self (Greenwald, 1980). More specifically, when being asked to describe one’s own personality, participants typically assign themselves more positive than negative personality adjectives (Alicke, 1985; Kwan *et al.*, 2007; Zhang *et al.*, 2013). This effect is accompanied by shorter RTs to positive self-descriptive words as compared to negative self-descriptive words (Watson *et al.*, 2007). This positivity bias is also reflected in memory processes, as the recall of positive personal information is much easier and more efficient than the recall of negative personal information (Kuiper and MacDonald, 1982). In addition, positive self-face evaluation is associated with the activation of posterior parts of the cingular cortex, a brain region that varies in activity with arousal state (Leech and Sharp, 2014) and is correlated with self-esteem measures (Oikawa *et al.*, 2012). The self-positivity bias is quite robust and has been obtained across a diverse representation of samples, varying in age, gender, psychopathology and culture (Brown and Kobayashi, 2002; Sedikides *et al.*, 2003; Mezulis *et al.*, 2004). However, in most cases positive self-association occurs unconsciously or in an implicit mode (Greenwald and Banaji, 1995; Jones *et al.*, 2002). While the self-positivity bias refers to many self-related domains, the IPA theory is focused on self-face processing. Its key assumption is that an IPA with the self mediates its advantage in face recognition, i.e. the process of recognizing one’s own face activates positive attributes in the self-concept, which facilitates responses to the self-face and thus results in a self-advantage in face recognition.

However, if saliency of the self-face is the primary driving factor of prioritized processing, it would imply a similar processing of the self-face and other salient faces, i.e. fearful faces. Fearful faces (emotive social stimuli) that effectively capture our attention (Troiani *et al.*, 2014) are processed with priority and have a privileged access to awareness (Stein *et al.*, 2014). This is also the case for the self-face (Wójcik *et al.*, 2018, 2019). For these reasons, it is possible that faces sharing such an extreme saliency feature could be processed similarly at the neural level. Therefore, in the current electroencephalogram (EEG) study, the processing of the self-face and emotionally positive and emotionally negative faces was investigated. In addition, neutral faces were introduced as control stimuli. This allowed us to address the question of whether the effects observed for the self-face and emotional faces can be explained by the saliency of faces in general. The task was a simple detection of the mentioned stimuli.

The analysis of ERPs was focused on ERP components commonly reported in studies with self-referential and/or emotional stimuli: P3—a positive ERP component occurring around 300 ms after the stimulus onset, with its maximum over central–parietal scalp sites (Tacikowski and Nowicka, 2010), and the late positive potential (LPP)—a positive, sustained ERP component starting around 500 ms after stimulus onset with a wide (frontal–central–parietal) topography (Kotlewska and Nowicka, 2016; Grecucci *et al.*, 2019). The functional role of P3 is associated mainly with attentional resource allocation (Polich, 2007). Increased P3 amplitudes have been found for both the self-face (e.g. Tacikowski and Nowicka, 2010; Kotlewska and Nowicka, 2015) and emotional faces (e.g. Luo *et al.*, 2010). Enhanced LPP, in turn, has most often been reported in studies investigating the processing of emotional and neutral faces (e.g. Schupp *et al.*, 2004; Herbert *et al.*, 2013; Zhang *et al.*, 2018), but it was also observed in the case of self-face processing (Zhong *et al.*, 2016). LPP reflects a spatially non-specific (i.e. global) temporary increase in attention that serves to facilitate the processing of the affective stimulus that elicited the LPP (Brown *et al.*, 2012).

We hypothesized that P3 and LPP to the self-faces would be significantly enhanced in comparison to neutral faces. As far as the relation between the self-face and emotionally positive and negative faces is concerned, we did not have any specific a priori expectations about the direction of the effect. Thus, we aimed at exploring this issue using different methods of EEG data analysis in addition to ERPs.

Hence, the collected EEG data were also analysed using a data-analytical framework called representational similarity analysis (RSA; Kriegeskorte *et al.*, 2008). RSA enables abstracting from the activity patterns themselves. Instead, multi-channel measures of neural activity are quantitatively related to each other and to a computational theory by comparing representational dissimilarity matrices that characterize the information carried by a given representation in a brain or model. As emotional faces and the self-face may elicit distinct spatial patterns of activity, a method that allows us to probe the EEG for similarities/dissimilarities in distributed neuronal codes complements the standard univariate approach. More specifically, RSA is a multivariate approach that accesses distributed information that would normally be lost through averaging procedures. In addition, it allows to test models in which variables can overlap or are represented in distinct states. Taken as a whole, this suggests that this method is perfectly suited for comparing the neuronal correlates of self-face processing and the processing of emotional faces in order to establish plausible commonalities in the spatial distribution of activity.

In addition to the similarity/dissimilarity metric obtained by applying RSA, the distinct spatial patterns of activity elicited by different types of faces were also tested with spatio-temporal cluster-based permutation tests (Maris and Oostenveld, 2007). This method enables unbiased comparisons of EEG signal recorded in different experimental conditions at all sensors and all time points while controlling for multiple comparisons and maximizing power by employing the cluster structure of the data as its sole test statistic. We used this approach to test for differences in spatial and temporal distributions between experimental conditions. Altogether, ERP, RSA and permutation test findings complement each other, providing a global and complete view of commonality/distinctiveness in the neural underpinnings of self-face and emotional faces processing.

It is worth noting that this approach, i.e. using different methods of EEG data analysis, can be seen in the context of the multiverse analysis approach (Steegen *et al.*, 2016). It has been argued that going beyond a single analysis of the experimental data should become a standard practice, and instead of analysing the data set with one method, researchers should perform multiple analyses on the same data set. In this way, findings obtained in one type of analysis could be confronted with findings from different methods, thus confirming (or undermining) conclusions drawn from the initial analysis.

## Materials and methods

### Participants

Thirty participants (16 females and 14 males) between the ages of 20 and 33 (*M* = 26.033; s.d. = 3.045) took part in the study. All participants were right-handed as verified with the Edinburgh Handedness Inventory (Oldfield, 1971). Only participants with normal or corrected-to-normal vision with the use of contacts and with no distinctive facial marks were recruited. This restriction was introduced to ensure the uniformity of visual stimuli standards, as the photograph of every participant was matched with photographs from the Karolinska Directed Emotional Faces (KDEF) database (Lundqvist *et al.*, 1998). Images included in this database present faces without glasses and without any visible marks. All participants reported no history of mental or neurological diseases. The required sample size was estimated using the G*Power 3 software (Faul *et al.*, 2007). The analysis was conducted for a one-way repeated-measures analysis of variance (ANOVA) with four measurement levels (estimated effect size *f* = 0.25, α = 0.05, β = 0.90, and non-sphericity correction ε = 1.0). It yielded a sample size of 30 participants. One data set, however, had to be excluded from the sample during preprocessing based on a technical malfunction.

### Ethics statement

The human ethics committee of the SWPS University of Social Sciences and Humanities (Warsaw, Poland) approved the experimental protocol. Written informed consent was obtained from each participant prior to the study and all participants received financial compensation for their participation.

### Stimuli

In the current study, similar to our previous studies on the topic of self-face processing, the set of stimuli was individually tailored for each participant (Tacikowski and Nowicka, 2010; Tacikowski *et al.*, 2011; Cygan *et al.*, 2014; Kotlewska and Nowicka, 2015; Kotlewska *et al.*, 2017; Wójcik *et al.*, 2018, 2019). It consisted of single face images of four types: the self-face, an emotionally negative (fearful) face, an emotionally positive (happy) face and a neutral face. Self-face photographs were taken prior to the experiment. All participants were invited to the lab to have a photograph of their face taken in a standardized environment (the same background and lightning conditions). Participants were asked to maintain a neutral facial expression when photographed. Photographs of emotional and neutral faces were taken from the A or B series of the KDEF database (Lundqvist *et al.*, 1998). To ensure that neutral and emotional facial expressions were recognized, we selected actors on the basis of the unbiased hit rates of detection (Goeleven *et al.*, 2008). The gender of faces from the KDEF database was matched to each subject’s gender in order to control for the between-category variability. Different images of emotional and neutral faces were used in individual sets of stimuli in order to avoid the plausible influence of one selected image on a pattern of brain activity. In each stimuli set, the KDEF images represented three different identities, i.e. if an image of a happy face of a given actor was selected, the images of fearful and neutral faces came from two different actors. Pictures within each stimuli set (i.e. the self-face image and selected KDEF images) were extracted from the background, grey-scaled, cropped to include only the facial features (i.e. the face oval without hair), resized to subtend 6.7° × 9.1° of visual angle and equalized for mean luminance using Photoshop® CS5 (Adobe, San Jose, CA). We did not normalize contrast and spatial frequencies in the pictures as these procedures tend to introduce substantial distortions into processed images. They were presented against a black background. None of the stimulus was shown to the participants before the experiment. The image of each participant’s face was removed from the computer disc at the end of the experimental session.

### Procedure

Participants were seated comfortably in a dimly lit and sound-attenuated room with a constant viewing distance of 57 cm from the computer screen (Eizo Flex Scan EV-2450, Hakusan, Ishikawa, Japan). After electrode cap placement (ActiCAP, Brain Products, Munich, Germany), the participants used an adjustable chinrest to maintain a stable head position. Presentation software (Version 18.2, Neurobehavioral Systems, Albany, CA) was used for stimuli presentation. Participants completed a simple detection task, regardless of the image presented (self-face, emotional or neutral face), and the participants were asked to push the same response button (Cedrus response pad RB-830, San Pedro, USA) as quickly as possible. After reading the instructions displayed on the screen, participants initiated the experiment by pressing a response button. Each trial started with a blank screen, shown for 1500 ms. Next, a white cross (subtending 0.5° × 0.5° of visual angle) was centrally displayed for 100 ms and then followed by a blank screen lasting either 300, 400, 500 or 600 ms at random. Subsequently, a stimulus was presented for 500 ms, followed by a blank screen for 1000 ms. The number of repetitions for each face category was 72. The order of stimuli presentation was pseudo-randomized, i.e. no more than two stimuli of the same category were displayed consecutively. A break was planned in the middle of experiment to keep participants from tiring. It lasted 1 min, unless the participant decided to start the second part of the experiment earlier. Participants needed on average 19 min to complete the whole experiment.

### EEG recording

The EEG was continuously recorded with 62 Ag–AgCl electrically shielded electrodes mounted on an elastic cap (ActiCAP, Brain Products, Munich, Germany) and positioned according to the extended 10–20 system. Two additional electrodes were placed on the left and right earlobes. The data were amplified using a 64-channel amplifier (BrainAmp MR plus; Brain Products, Germany) and digitized at a 500-Hz sampling rate, using BrainVision Recorder software (Brain Products, Munich, Germany). EEG electrode impedances were kept below 10 kΩ. The EEG signal was recorded against an average of all channels calculated by the amplifier hardware.

### Behavioural analysis

Responses within a 100–1000 ms time window after stimulus onset were analysed using SPSS (Version 26, IBM Corporation) and JASP (Wagenmakers *et al.*, 2018) software packages. A Shapiro–Wilk test for normality conducted on the distribution of RTs for each stimulus type (self-face, emotionally positive face, emotionally negative face and neutral face) revealed that the distribution of RTs deviated from normality for two stimulus types. Therefore, a Friedman test was used with type of stimulus (self-face, emotionally positive face, emotionally negative face and neutral face) as a within-subject factor. The results are reported with reference to an α-level equal to 0.05.

To conduct statistical analyses of behavioural (RT) and ERP data in a consistent manner, similar to our analyses of ERP components, the traditional null hypothesis significance testing approach was complemented with Bayesian analysis methods. Bayes factors (BFs) were computed using JASP software (Wagenmakers *et al.*, 2018). A BF_10_ between 1 and 3 implies anecdotal evidence for the presence of an effect (i.e. anecdotal evidence for H_1_). A BF_10_ between 3 and 10 gives moderate evidence, a BF_10_ between 10 and 30 indicates strong evidence for the presence of an effect, BF_10_ between 30 and 100—very strong evidence, and a BF_10_ higher than 100—extreme evidence for H_1_ (Lee and Wagenmakers, 2014).

### ERP analysis

Offline analysis of the EEG data was performed using BrainVision Analyzer® software (Version 2.2, Brain Products, Gilching, Germany). EEG data from 62 channels were re-referenced offline to the algebraic average of the signal recorded at the left and right earlobes, notch-filtered at 50 Hz, and band-pass-filtered from 0.01 to 30 Hz using a second-order Butterworth filter. After re-referencing and filtering the signal, ocular artefacts were corrected using Independent Component Analysis—ICA (Bell and Sejnowski, 1995). After the decomposition of each data set into maximally statistically independent components, components representing eye blinks were rejected based on a visual inspection of the component’s topography (Jung *et al.*, 2001). Using the reduced component-mixing matrix, the remaining ICA components were multiplied and back-projected to the data, resulting in a set of ocular-artefact-free EEG data. Subsequently, the EEG signal was segmented into 1700-ms-long epochs, from −200 ms before to 1500 ms after stimulus onset. The next step was a semi-automatic artefact rejection procedure that rejected trials exceeding the following thresholds: the maximum permitted voltage step per sampling point was 50 µV, the maximum permitted absolute difference between two values in the segment was 200 µV and the lowest permitted activity within a 100-ms interval was 0.5 µV. The mean number of segments that were averaged afterwards for each category of stimuli was as follows: self-face—72.241 (s.d. = 2.430), emotionally positive face—72.414 (s.d. = 1.991), emotionally negative face—71.621 (s.d. = 2.624) and neutral face—72.172 (s.d. = 1.910). The number of epochs used to obtain ERPs did not differ significantly between the types of stimuli. Finally, the epochs were baseline-corrected by subtracting the mean of the pre-stimulus period.

**Fig. 1.**
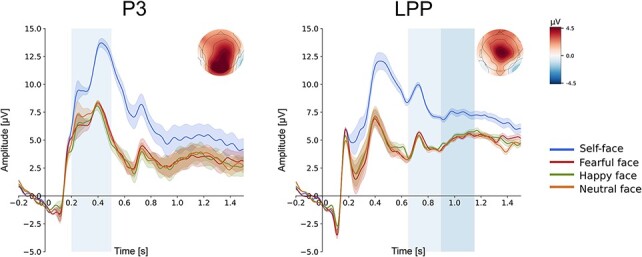
Grand average ERPs to self-face, fearful, happy and neutral faces. Shaded areas indicate standard deviations (s.d.). Left panel: P3 component for pooled electrodes PZ, CPZ, CP2 and P2 that are within the region of maximal activity in the topographical distribution of brain activity, averaged across all experimental conditions (i.e. four types of faces). Right panel: LPP for pooled electrodes FCZ, FC2 and C2 that are within the region of maximal activity in the topographical distribution of brain activity, averaged across all experimental conditions (i.e. four types of faces). The analysed time windows are marked by light-blue rectangles.

Selection of electrodes for ERP analyses has to be orthogonal to potential differences between experimental conditions (Kriegeskorte *et al.*, 2009). Therefore, this has to be done on the basis of the topographical distribution of brain activity (in the time window corresponding to a given component) averaged across all experimental conditions. Electrodes lying within the maxima identified in such a topographical map should be further analysed. Based on the topographical distribution of activity as well as grand-averaged ERPs, collapsed for all experimental conditions (self-face, emotionally positive face, emotionally negative face and neutral face), the following windows were chosen for analysis of ERP components of interest: 200–500 ms for P3 and 650–900 ms and 900–1150 ms for LPP (Figure 1). Two clusters of electrodes within the region of maximal activity were selected: (1) for P3—PZ, CPZ, CP2 and P2 and (2) for LPP—FCZ, FC2 and C2. The data were pooled for those electrodes. This step is justified by the limited spatial resolution of EEG and high correlation between neighbouring electrodes. The mean values at each time point within the aforementioned time windows were used to assess the amplitudes of our ERP components of interest. This method is less affected by possible low signal-to-noise ratio than peak measure methods (Luck, 2005).

All statistical analyses were performed using SPSS software (Version 26, IBM Corporation), custom Python scripts (Version 3.5, Python Software Foundation) and JASP software (Wagenmakers *et al.*, 2018). The Shapiro–Wilk normality test was conducted on P3, LPP (650–900 ms) and LPP (900–1150 ms) amplitude distributions. For P3 and LPP (650–900 ms) they did not deviate from normality, thus a one-way repeated-measures ANOVA was performed with category of stimuli (four levels: self-face, emotionally positive face, emotionally negative face and neutral face) as a within-subject factor. For LPP (900–1150 ms) with a non-normal amplitude distribution, a Friedman test was applied analogously. Thus, a one-way repeated-measures ANOVA was performed with category of stimuli (four levels: self-face, emotionally positive face, emotionally negative face and neutral face) as a within-subject factor. All effects with more than one degree of freedom in the numerator were adjusted for violations of sphericity (Greenhouse and Geisser, 1959). Bonferroni correction for multiple comparisons was applied to *post*  *hoc* analyses. All results are reported with α-levels equal to 0.05.

The traditional null hypothesis significance testing approach was complemented with Bayesian analysis methods. To test whether the self-face and other faces were characterized by similar levels of neural activity, BFs were computed using JASP software (Wagenmakers *et al.*, 2018). The main reason for choosing BF was that, unlike classic frequentist statistics, BF evaluates how strongly both alternative and null hypotheses are supported by the data. Specifically, BF is a ratio of the probability (or likelihood) of observing the data given the alternative hypothesis is true to the probability of observing the data given the null hypothesis is true. Thus, in our particular case, BF provides further evidence either in favour of similarities or rather differences in self-face and emotional faces processing. The medium prior scale (Cauchy scale 0.707) was used in all Bayesian tests. The Results section provides interpretations of the BF_10_ according to Lee and Wagenmakers (2014).

### Cluster-based permutation tests

Cluster‐based permutation tests were used here as an exploratory analysis procedure, as they efficiently handle the multiple comparisons problem in high‐dimensional magnetoencephalographic and EEG data (Sassenhagen and Draschkow, 2019). In contrast to the ERP method, which focuses on data recorded at a single electrode or small set of electrodes in a specific time window, cluster-based permutation tests allow for EEG signal amplitude across all electrodes and all time samples to be compared. We directly compared: self-face *vs* fearful face processing, self-face *vs* happy face processing and self-face *vs* neutral face processing. As clustering in both space and time was used, such an analysis procedure revealed differences in the spatial distributions of activity as a function of time between the tested conditions.

In general, permutation tests are used to test the null hypothesis that the data in the experimental conditions come from the same probability distribution. Getting a significant result means that the null hypothesis can be rejected in favour of the alternative hypothesis, i.e. that the data came from different distributions. Therefore, significant results from permutations tests indicate a significant between-condition difference. The results are reported with reference to an α-level equal to 0.05.

The calculation of a cluster-based permutation test for multiple sensors is performed in the following steps: (i) for every sample, the EEG signal is compared between the two conditions by means of a *t*-value, (ii) all samples whose *t*-values are larger than a threshold [in our study we used the threshold-free cluster enhancement (TFCE) method] are selected, (iii) the selected (sensor, time) samples are clustered on the basis of spatial and temporal adjacency, (iv) cluster-level statistics are calculated by taking the sum of the *t*-values within a cluster and (v) finally, the largest of the cluster-level statistics is taken. The TFCE eliminates the free parameter initial threshold value that determines which points are included in clustering by approximating a continuous integration across possible threshold values with a standard Riemann sum. A significant advantage of TFCE is that, rather than modifying the null hypothesis under testing, it modifies the data under testing while still controlling for multiple comparisons. The statistical test is then done at the level of individual voxels rather than clusters. This allows for the significance of each point to be evaluated independently rather than only as cluster groups.

The non-parametric statistical test is performed by calculating a *P*-value under the permutation distribution and comparing it with some critical α-level (0.05 in our study). The permutation distribution is obtained by the following procedure: (i) the trials of the two experimental conditions in a single set are collected, (ii) the trials are randomly partitioned into two subsets, (iii) the test statistics is calculated on this random partition and (iv) steps (ii) and (iii) are repeated a large number of times and a histogram of the test statistics is constructed. In practice, it is not possible to calculate the permutation *P*-value by repeating steps (ii) and (iii) an infinite number of times. Instead, this *P*-value is approximated by a so-called Monte Carlo estimate. This Monte Carlo estimate is obtained by repeating steps (ii) and (iii) a large number of times and comparing these random test statistics (i.e. draws from the permutation distribution) with the observed test statistics. The Monte Carlo estimate of the permutation *P*-value is the proportion of random partitions in which the observed test statistics is larger than the value drawn from the permutation distribution. The accuracy of the Monte Carlo *P*-value increases with the number of draws from the permutation distribution. In our study, the Monte Carlo *P*-values were calculated on 1000 random partitions.

Cluster-based permutation tests were conducted using custom-made Python scripts with use of the mne.stats.spatio_temporal_cluster_1samp_test function from the MNE Python package.

### Representational similarity analysis (RSA)

#### Representational geometry.

A representation of an experimental condition in geometrical space can be defined as a point or cloud of points in a multidimensional space (Kriegeskorte and Kievit, 2013). When analysing EEG signals, these dimensions can be thought as the electrical activity recorded by separate electrodes. The geometrical relation of two neuronal responses can be analysed through the comparison of their locations within this ‘electrode’ space. That is, a metric such as Euclidean or Mahalanobis distance between these responses in multidimensional space is computed. Euclidean distance was used in the present study to transform the data into geometrical space (Kriegeskorte *et al.*, 2006). Such an approach provides a detailed account of the geometrical structure formed by distinct conditions. It can reflect differences, similarities, and even how much variance in these comparisons is explained by an external factor.

#### RSA template-based regression.

To calculate the Euclidean distance matrices, we first calculated the mean epochs for each condition for each subject. Then, 8 × 8 Euclidean distance matrices (2 sets of data × 4 conditions) were computed for each time point, yielding an 851 × 8 × 8 matrix for each participant. To improve the sensitivity of the method, the distance matrix was enlarged by subsampling every condition. More specifically, the trials within each condition were randomly assigned into two pools. This resulted in an 8 × 8 distance matrix. Next, we applied a least-squares multiple regression model to assess the contribution of the predicted ‘template’ neuronal codes to the distance matrix:


$$\begin{equation*}D = {\beta _0} + \mathop \sum \limits_{n = 1}^3 {\beta _n}templat{e_n} + {\rm{\varepsilon }}\end{equation*}$$

where D denotes the distance matrix obtained from RSA and ε denotes the error (residual) of the model. β_0_ denotes the intercept of the model, which was coded as the identity matrix of the same dimensions as the template matrices. The three template matrices (templates), indexed by the counter (*n*) were regressed onto the distance matrix to obtain the corresponding regression weights (β_0–3_). β-values indicate the relative contribution of each template matrix (regressor) to the variance in the distance matrix. The three predicted template matrices were as follows: (i) self-face and emotionally negative face are similar and differ from the two other faces (‘self-face + fearful face model’), (ii) self-face and emotionally positive face are similar and differ from the two other faces (‘self-face + happy face model’) and (iii) self-face differs from all other faces (‘self-face model’). These templates were then converted to *z*-scores to allow for comparisons. The output of our model was a matrix containing β-values for every person, for every time point, for every regressor (29 × 851 × 3). Furthermore, the resulting β-values were temporally smoothened using a Gaussian window with a width of 32 ms.

## Results

### Behavioural results

The mean number of responses to all types of stimuli were as follows (mean ± standard error): self-face (71.500 ± 0.755), fearful face (71.321 ± 0.568), happy face (71.786 ± 0.581) and neutral face (71.679 ± 0.385). Differences between the numbers of responses for different types of faces were non-significant.

The RTs of one participant were found to be greater than 3 s.d. above the mean for each condition, and they were subsequently excluded from further behavioural analysis. A repeated-measures ANOVA, conducted on median RTs in the group of 28 participants, revealed a significant effect of type of stimuli: *F*_3,81_ = 3.576, *P* = 0.0174, η^2^ = 0.117. *Post* *hoc* comparisons showed that RTs to self-face were significantly shorter than to fearful face (*P* = 0.014, BF_10_ = 16). Participants also reacted faster to self-face than to happy face; however, it was only a statistical trend (*P* = 0.087, BF_10_ = 3). The other comparisons were non-significant. Descriptive statistics are shown in Table 1.

**Table 1.** Mean median RTs and standard deviation (s.d.) for each type of stimuli (*N* = 28)

	Mean medians	s.d.
Self-face	244.925	26.259
Fearful face	248.754	25.678
Happy face	249.268	25.387
Neutral face	247.652	24.743

### ERPs results

Mean P3 and LPP amplitudes and s.d. values for correct trials were computed for each type of stimulus, i.e. self-face, fearful face, happy face and neutral face (see Table 2). Grand-average ERPs for all types of faces are presented in Figure 1.

**Table 2.** Mean (M) amplitude (μV) and standard deviation (s.d.) for each analysed component (*N* = 29)

	P3		LPP (650–900 ms)		LPP (900–1150 ms)	
	*M*	s.d.	*M*	s.d.	*M*	s.d.
Self-face	7.771	3.545	16.789	9.129	15.146	9.101
Fearful face	5.103	2.694	9.078	8.033	10.296	8.245
Happy face	4.811	2.262	9.010	8.276	10.938	8.394
Neutral face	5.300	2.220	8.759	7.853	10.399	7.744

#### P3 (250–500 ms).

One-way repeated-measures ANOVA showed a significant main effect of stimulus type: *F*_3,84_ = 31.500, *P* < 0.0001, η^2^ = 0.529. *Post*  *hoc* analyses revealed that P3 amplitude to the self-face was significantly higher than P3 amplitudes to fearful (*P* < 0.0001, BF_10_ = 10 038), happy (*P* < 0.0001, BF_10_ = 78 764) and neutral faces (*P* < 0.0001, BF_10_ = 3 046). All other comparisons were non-significant.

#### LPP (650–900 ms).

One-way repeated-measures ANOVA revealed a main effect of stimulus type: *F*_3,84_ = 50.332, *P* < 0.0001, η^2^ = 0.643. In an early time window, LPP amplitude to the self-face was significantly higher than that to fearful (*P* < 0.0001, BF_10_ = 517 949), happy (*P* < 0.0001, BF_10_ = 2.842 × 10^6^) and neutral faces (*P* < 0.0001, BF_10_ = 7.625 × 10^6^). All other comparisons were non-significant.

#### LPP (900–1150 ms).

A Friedman test yielded a statistically significant difference between LPP amplitudes in the later time window for stimuli type: χ^2^ (3) = 21.290, *P* < 0.001. For *post*  *hoc* analyses, Wilcoxon signed-rank tests with Bonferroni correction (significance level set at *P* < 0.01) were used. These comparisons revealed significantly higher LPP amplitude to the self-face than to fearful (*Z* = −4.141, *P* < 0.0001, BF_10_ = 1 142), happy (*Z* = −3.449, *P* < 0.001, BF_10_ = 134) and neutral (*Z* = −4.033, *P* < 0.0001, BF_10_ = 1 748) faces. All other comparisons were non-significant.

### Cluster-based permutation tests

**Fig. 2.**
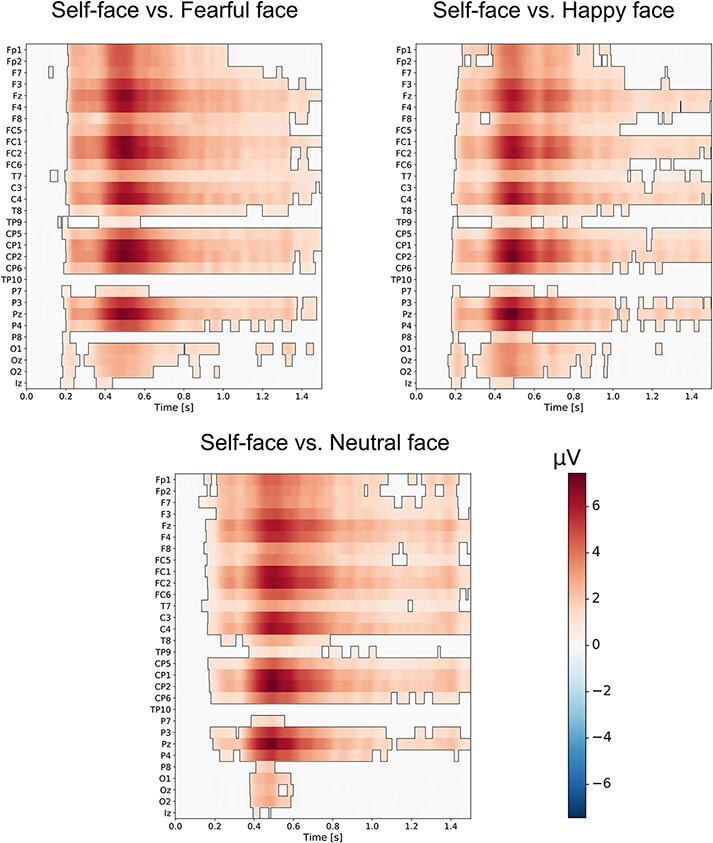
Results of cluster-based permutations tests. Self-face was compared to fearful and happy face (top left and top right panels, respectively) as well as to neutral face (bottom panel). Statistically significant positive differences between tested experimental conditions are indicated in red (*P* < 0.05). For illustrative purposes, 30 electrodes from the set of 62 are presented. The remaining 32 electrodes are presented in Figure S2 in the supplementary material.

The results of our cluster-based permutation tests indicated that self-face processing differed significantly from the processing of happy, fearful and neutral faces. The differences between those experimental conditions were widely distributed in space and time. They started around 200 ms after the visual stimulus onset and lasted for the subsequent 1200–1400 ms. They were present at numerous electrode sites in the frontal, central and parietal regions. The cluster-based permutation results are presented in Figure 2 for 30 of 62 analysed electrode sites (Figure S2 in the supplementary material shows the results of cluster-based permutation tests for the remaining 32 electrode sites). It is interesting that the broad time window of substantial differences between tested conditions encompasses the time windows in which both ERP components were analysed (250–500 ms and 650–1150 ms for P3 and LPP, respectively). In addition, although P3 and LPP were analysed at electrode sites that were selected on the basis of maximal activity in the topographical distribution maps, similar effects (i.e. higher amplitudes of these ERP components to the self-face than to other faces) were present at virtually all electrodes (Supplementary material S1).

### RSA results

**Fig. 3.**
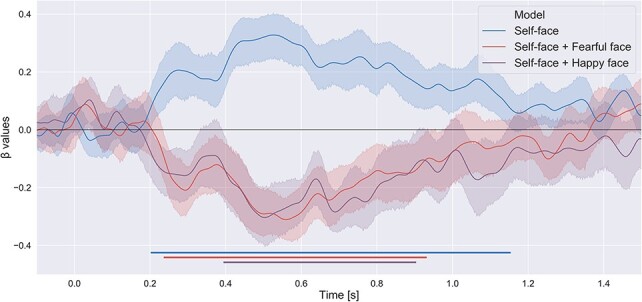
Results of the representational similarity analysis. Shaded areas indicate confidence intervals (CIs). Three models were tested: (i) self-face and fearful face differ from other faces (happy and neutral); (ii) self-face and happy face differ from other faces (fearful and neutral) and (iii) self-face differs from all other faces (fearful, happy and neutral). Cluster-based one-sample *t*-tests revealed significant effects for all models (α-levels below 0.05 are indicated by horizontal blue, red and violet lines parallel to the *x*-axis). However, the first two were negative, not positive, predictors.

Three different models were computed and tested. The first two models were based on the assumption of similarities in the distribution of neural activity associated with the (i) self-face and fearful face (‘self-face + fearful face model’) and (ii) the self-face and happy face (‘self-face + happy face model’). The third model assumed a unique distribution of activity in the case of self-face processing (‘self-face model’) that did not resemble (i.e. was dissimilar from) distributions of activity for all other faces (happy, fearful and neutral). Thus, similarities in the distribution of neural activity for different experimental conditions implies that the neural code corresponding to the representations of those conditions is similar. Cluster-based one-sample permutation *t*-tests revealed that the model assuming a similarity structure between the distributed patterns of activity elicited by the self-face and the fearful face is a negative predictor of the neuronal activity (cluster time points: 236–932 ms, *P* < 0.001). This suggests that the topographies observed in the self-face and fearful face conditions became more dissimilar as a function of time, starting from an early period of the trial. A similar result was found in the case of the model assuming a similarity structure between the happy and self-face (cluster time points: 394–904 ms, *P* < 0.001). The third model aimed to capture a dissimilarity structure between the self-face and every other experimental condition, as well as a similarity structure between the fearful, happy and neutral faces. A cluster-based permutation test revealed that this model is a positive predictor of the neuronal activity (cluster time points: 202–1154 ms, *P* < 0.001). That is, the spatially distributed pattern of activity elicited in the self-face condition becomes dissimilar to the patterns elicited by other experimental conditions early on in the trials, and this dissimilarity increases as a function of time. This is in line with the first two models and suggests a distinct processing pipeline between the self-face and other experimental conditions. Figure 3 illustrates these results.

## Discussion

Despite the fact that recent years have seen a substantial increase of interest in the self in various disciplines, leading to the publication of multiple papers on the topic, many questions still remained unanswered. One of them refers to the factors that determine the prioritized self-face processing that has been well-documented in numerous studies with different experimental approaches (for a review see: Humphreys and Sui, 2016). As humans are the subject of their own cognition, they are in the unique position of possessing years of detailed visual, tactile, motor and sensory-feedback experiences about themselves, which results in a highly elaborated (not only visual but also multimodal) representation of their own image (Li and Tottenham, 2013). The special saliency of the self-face has been largely agreed upon (Lavie *et al.*, 2003; Gray *et al.*, 2004; Brédart *et al.*, 2006; Pannese and Hirsch, 2011), and converging lines of evidence have confirmed the special status of self-face processing (Bortolon and Raffard, 2018).

The current study aimed at elucidating the plausible role of an emotional relevance factor in the preferential processing of this stimulus by direct comparisons between the self-face and emotional as well as neutral faces. Two types of emotional faces were used: (i) happy faces were introduced, motivated by the self-positive bias (e.g. Greenwald, 1980; Watson *et al.*, 2007) and (ii) fearful faces, because of their high perceptual saliency, i.e. a feature shared with one’s own face (Elsherif *et al.*, 2017). EEG data were collected while participants performed a simple detection task. The obtained data were analysed using three methods that complement each other: ERP component amplitude analysis, RSA and cluster-based permutation tests.

The results obtained using those methods clearly indicate that the processing of the self-face substantially differed from the processing of all other (emotional and neutral) faces. Specifically, the process of self-face detection was associated with substantially increased P3 and LPP amplitudes in comparison to emotionally positive, emotionally negative and neutral faces. These effects were both highly significant and robust (mean amplitudes to the self-face were about two times higher than to other faces, either emotional or neutral). In addition, BF_10_ values for comparisons between amplitudes of the analysed ERP components elicited by the self-face and other faces indicated extreme evidence in favour of the alternative hypothesis (all BFs_10_ > 100). P3 and LPP topography indicated maximal regions of activity in the parietal–central and frontal regions mainly in the right hemisphere. This is in line with fMRI findings indicating the involvement of the right hemisphere (in particular, right fronto-parietal structures) in visual self-recognition (e.g. see Hu *et al.*, 2016 for review; Keenan *et al.*, 2000).

The results of the RSA and cluster-based permutation tests revealed differences between self-face processing and the processing of other types of faces. The RSA that assessed the similarity/dissimilarity of neural activity patterns elicited by the self-face and emotionally positive face, as well as by the self-face and emotionally negative faces, definitely showed that they were highly dissimilar. Thus, RSA findings in the current study strongly point to differences in the spatial distribution of neuronal activity between the processing of self-face and emotional faces. Moreover, cluster-based permutation tests, which were used to contrast the self-face and emotionally positive faces as well as the self-face and emotionally negative faces, indicated strong and significant differences between the tested conditions. Altogether, the results of different methods used to test similarities between the processing of self-face and happy faces as well as self-face and fearful faces indicate that their neural correlates substantially differed. Importantly, all of these results consistently show strong and significant differences between the self-face and other faces in a prolonged time window: they started 200 ms after the face onset and lasted till ca. 1200 ms.

Our results concerning long-lasting and sustained effects in self-face *vs* other faces discrimination are in line with the findings of other electrophysiological studies on self-face processing (Alzueta *et al.*, 2019, 2020). Specifically, it has been shown that the self-face is differentiated from other (familiar) faces as early as 200 ms (Alzueta *et al.*, 2019) and such differentiation continues until 1200 ms (Alzueta, 2020). The only difference between the aforementioned studies and the present study is the type of faces that served as a control condition to the self-face (familiar and unfamiliar neutral faces in Alzueta et al.’s studies; unfamiliar emotional faces in our study). Nevertheless, all those findings consistently pointed to sustained activity in the 200–1200 time window associated with the self-face as compared to different types of other faces.

One may argue that effects reported in the present study can be attributed to the extreme familiarity of the self-face in general, as the other types of faces (emotionally positive, emotionally negative and emotionally neutral) were unfamiliar to the participants. Therefore, one cannot rule out the possibility that the familiarity factor had an impact on the pattern of findings reported in the present study. However, the role of high familiarity in the preferential processing of any self-related stimuli has been questioned by numerous studies. Differences between self-face processing and the processing of other familiar faces (e.g. faces of celebrities) were reported in many studies (e.g. Tacikowski and Nowicka, 2010). Crucially, the role of familiarity seems to be challenged by findings of studies using highly familiar faces, i.e. the faces of close-others, as a control condition to the self-face. In general, they reported differences between the self and the close-other condition in favour of the self (Cygan *et al.*, 2014; Kotlewska and Nowicka, 2015; Kotlewska *et al.*, 2017). In those studies, the close-other was operationalized as the most important person at the time of experimentation and was freely chosen by each participant (e.g. a spouse, a partner and a very close friend). Nevertheless, differences between the self and the close-others’ faces were observed on the neural level as indicated by late ERP components (Cygan *et al.*, 2014; Kotlewska and Nowicka, 2015) and steady-state visual evoked potentials (Kotlewska *et al.*, 2017). This seems to indicate that the processing of even highly familiar faces, seen on an everyday basis, differs from the processing of the self-face.

An additional and very strong evidence against the role of the familiarity factor in the prioritized processing of self-related information comes from studies that aimed to investigate newly acquired self-related information (Sui *et al.*, 2012, 2014). It has been demonstrated that after being told to associate three identities (self, friend and stranger) with three arbitrary stimuli (geometrical shapes), participants were faster in a perceptual matching task at recognizing matching pairs of the self-associated shape with a label than for friend- or stranger-related pairings. It is worth noting that in those experimental paradigms levels of familiarity were equalized for the self and other conditions. The findings of this study provided evidence that a brief self-association is sufficient to facilitate processing of previously neutral and new stimuli with no relevance to the self.

However, as noted by Woźniak and Knoblich (2019), in the matching trials of the self-prioritization task, participants are processing not only self-associated arbitrary stimuli but also familiar verbal labels with a pre-experimentally established meaning. Therefore, the self-advantage may be caused by the familiarity of the labels, rather than the self-association of the shapes. Thus, in a recent study, Woźniak and Knoblich (2019) tested whether such self-prioritization can be observed in the absence of any pre-experimentally familiar stimulus related to the self. In their study, participants were asked to associate avatar faces with three identities (self, best friend and stranger). Afterwards, labels (you, friend and stranger) were replaced with unfamiliar abstract symbols that were associated with three identities before the actual experiment started. The results of that study presented the typical pattern of self-prioritization, showing that this effect does not critically depend on the presence of familiar labels and that it can be elicited by initially neutral stimuli. Altogether, those studies suggest that rapid and rather effortless association of initially neutral information with the self leads to subsequent prioritization of this information. All in all, the aforementioned findings undermine the role of the familiarity factor in eliciting the prioritized processing of self-related information.

Our P3 results corroborate the findings of previous studies reporting enhanced P3 to the self-face in comparison to other (either familiar or unfamiliar) faces (Ninomiya *et al.*, 1998; Scott *et al.*, 2005; Sui *et al.*, 2006; Tacikowski and Nowicka, 2010; Cygan *et al.*, 2014; Kotlewska and Nowicka, 2015). Moreover, the P3 results of the current study are in line with the findings of an earlier ERP study with self-face and emotionally negative faces presented as deviant stimuli in an odd-ball procedure (Zhu *et al.*, 2016). In that study, the amplitudes of P3 to the self-face were much higher than that to (unknown) emotional and neutral faces. In general, such patterns of P3 findings may be viewed in the context of classical models of face recognition (Bruce and Young, 1986; Burton *et al.*, 1990). Although in both studies (Zhu et al., 2016; the present study) the explicit recognition of faces was not required to successfully accomplish the behavioural tasks, it seems reasonable to assume that such recognition happened as it is a rather automatic and very fast process (Wójcik *et al.*, 2018).

Briefly, classical models of face recognition generally posit the following stages of this process: structural encoding, face recognition units (FRUs), person identity nodes (PINs) and semantic information units (SIUs). Structural encoding follows an initial pictorial analysis and consists in capturing the essential structural features of a face. If a face is known, it activates the FRU—a structural representation of a familiar face stored in long-term memory that takes into account the variability of viewpoints, changeable facial features, etc. Next, the corresponding PIN is activated, which is a multimodal representation of the face bearer. When the person is identified, biographical knowledge about them may also be retrieved. This retrieval is thought to entail activation of SIUs. Thus, the essential concept in this framework is the activation of the semantic information related to the identity of the recognized person, i.e. a specific and rich network of facts about the recognized individual (Burton *et al.*, 1990).

ERP studies carried out within the framework of the face recognition models linked the specific stages to specific ERPs components, with P3 reflecting access to PIN and SIU nodes (Paller *et al.*, 2000; Tacikowski *et al.*, 2011). Thus, substantially increased amplitudes of P3 to self-face presentations may result from the extremely rich semantic information referring to the self. Importantly, this type of information is absent in the case of emotional and neutral faces that were unfamiliar to participants, and for that reason no semantic information was available. This may explain both the significant differences between P3 amplitudes to images of the self-face and other faces, as well as the lack of P3 differences between emotional and neutral faces observed in the present study. It is worth noting that in previous studies the amplitude of the P3 component differed as a function of emotional expression (e.g. Cuthbert *et al.*, 2000; Keil *et al.*, 2002; Schupp *et al.*, 2004; Briggs and Martin, 2009; Foti *et al.*, 2009). The lack of these differences in our experiment suggests that the activation of the semantic network related to the self may overwrite earlier saliency effects, i.e. different sources of saliency can interact with each other.

However, other interpretations of P3 findings are also plausible. It is worth noting that the current debate on the functional role of the P3 component refers to many different topics. Among them is the theoretical framework proposing that the P3 reflects the response of the neuromodulatory locus coeruleus–norepinephrine (LC–NE) system to the outcome of internal decision-making processes and the consequent effects of noradrenergic potentiation of information processing (Nieuwenhuis *et al.*, 2005). It was also suggested that P3 may reflect reactivation of well-established stimulus–response (S–R) links (Verleger *et al.*, 2015). Nevertheless, in the context of the present study, P3 interpretations referring to attentional processes seem to be most relevant. Specifically, it has been proposed that the mechanisms boosting the prioritized processing of self-relevant information could be driven by automatic capture of attention and prioritized allocation of attention to the self-related stimuli (review: Humphreys and Sui, 2016; Sui and Rotshtein, 2019). Indeed, several studies found that the self-face automatically captures attention (e.g. Tong and Nakayama, 1999; Brédart *et al.*, 2006; Alexopoulos *et al.*, 2012; Alzueta *et al.*, 2020), and numerous EEG studies have revealed greater P3 amplitude in response to one’s own face (e.g. Tacikowski and Nowicka, 2010; Ninomiya *et al.*, 1998; Sui *et al.*, 2006; review: Knyazev, 2013). As P3 is usually associated with attentional processes (for review see: Polich, 2007), our P3 findings indicate preferential engagement of attentional resources to the self-face. Such an interpretation suggests that the preferential processing of the self as reflected by the P3 may be caused by an early allocation of attentional resources and not a late attentional facilitation caused by a semantic activation (as proposed by the face recognition model). This notion seems to be further supported by the central–parietal topography of the P3 (Polich, 2007). At this point, it should be stressed that reported pattern of findings is not likely to be driven by decision-making processes (there was no specific decision to be made, just a simple detection of a stimulus) or S–R links (regardless of seen face, participants always were pressing the same button).

However, not only P3 but also LPP was significantly increased in the self-face condition. LPP is typically increased by emotional stimuli when compared to neutral visual stimuli (Cuthbert *et al.*, 2000; Hajcak and Nieuwenhuis, 2006; Foti and Hajcak, 2008; Olofsson *et al.*, 2008) and reflects enhanced processing and attention to emotional salient stimuli (Cuthbert *et al.*, 2000). Larger LPP amplitudes are also correlated with increased arousal (Cuthbert *et al.*, 2000). The neural generators of LPP are thought to be the extrastriate visual system and emotion-related structures such as the amygdala (Sabatinelli *et al.*, 2007), and LPP may reflect stronger functional connectivity between the occipital cortex and frontal areas for high arousing emotional relative to low arousing neutral stimuli (Moratti *et al.*, 2011). Our results do not reflect a pattern that was found in previous studies, i.e. the differences in LPP amplitude between emotional and neutral faces. Similar to the P3 component, only the self-face condition was characterized by an increase in LPP amplitude.

One of plausible explanations of this discrepancy may refer to findings of studies showing that the LPP can be modulated by reappraisal, with larger deflections when upregulating an emotional response (Moser *et al.*, 2009) and reduced deflections when downregulating an emotional response (Hajcak and Nieuwenhuis, 2006; Foti and Hajcak, 2008; Schönfelder *et al.*, 2014). One may speculate that the reported pattern of LPP findings (i.e. substantially enhanced LPP for the self-face and decreased LPP to all other faces) may be related to automatically elicited processes such as the augmentation of emotional response in the case of one’s own face and its reduction in the case of all other faces (emotional and neutral ones). An alternative interpretation of our findings may refer to the issue raised by Panksepp (1998, 2011): emotional feelings (rather than simple emotions) are intrinsically subjective. Thus, in contrast to (objectively) emotional faces, seeing the self-face may result in an emergence of subjective emotional states associated with increased brain activity. Therefore, the current findings may reflect the distinction between subjectively significant *vs* subjectively non-significant stimuli, with the self-face being a subjectively significant stimulus and all other faces being subjectively non-significant. This is in line with Bradley’s notion (2009) that the key stimulus dimension that modulates LPP amplitude is significance and that indicators of this construct include subjective ratings of arousal, autonomic response and the activation of specific neural circuits.

Importantly, the lack of P3 and LPP differences between emotional and neutral stimuli may be related not only to the early or late engagement of attentional resources. An additional and complementary explanation of that effect may refer to the degree or the magnitude of the saliency features of the presented faces and, therefore, to the specificity of these saliency effects. Both analysed ERP components are modulated by the saliency of stimuli (P3—Teixiero et al., 2010; LPP—Martin *et al.*, 2020) and thus, substantially increased P3 and LPP amplitudes to the self-face may reflect the extreme saliency of this stimulus, in line with other studies (Humphreys and Sui, 2015). However, P3 and LPP response to potentially salient emotional faces did not differ from P3 and LPP response to neutral faces. One may speculate that images of emotional faces were not viewed as salient when compared with the self-face image. Thus, it might be speculated that different sources of salience interact with each other (self-related *vs* not self-related) and exerted a differential influence on the analysed ERP components. Such a hypothesis seems to find some support in the results obtained by Marti et al. (Marti *et al.*, 2015; Marti and Dehaene, 2017). These authors showed that the processing of two different tasks or target stimuli can take place in parallel at early stages of information processing. However, at later stages the representations of each task/stimuli compete with each other for attentional resources where the winner is subject to an all-or-none activation. Although such an early parallel processing and late selection model seems to explain our P3 and LPP findings, it is worth noting that this model was tested using different experimental paradigms than those applied in the present study. In Marti et al.’s experiments, the stimuli were displayed in a rapid serial stream and their saliency was determined in a top-down fashion. It is yet to be determined whether events within a broader time scale can be subject to a similar processing architecture and how intrinsic saliency modifies these operations.

To further investigate the winner-takes-all late selection process, as revealed by the absence of P3 and LPP differences between emotional and neutral faces, additional analyses were conducted on the recorded data (the results of those analyses are included in the Supplementary Data). The analysis of an early face-selective ERP component (N170) revealed that both types of emotional faces differed from neutral faces. A linear discriminant analysis (LDA) conducted for a discriminant function between the happy, fearful and neutral faces clearly showed that these conditions were differentiated in an early time window. When the self-face condition was added, the decoder revealed that the category information persisted throughout the whole trial window (see Figure S4 and Figure S5 in the Supplementary Data). This is in line with the ERP analyses showing that the happy, fearful and neutral conditions are mainly differentiated early on, whereas the self-face condition adds a component that allows the information to persist in a late time window. These results suggest that the emotional saliency differentiated, in fact, the experimental conditions but only in an early time window. It seems that this saliency effect was overwritten by the special status of the self-face on later stages on information processing.

All in all, the findings of our different analytical approaches provide converging evidence of the self-face being processed preferentially at later stages of information processing. Moreover, this effect is unlikely to be caused by the low-level features of the images as the happy, fearful and neutral faces are differentiated by the participants in an early time window.

The aforementioned differences between the self-face *vs* other faces processing, observed at the neural level, were accompanied by differences at the behavioural level. Specifically, RTs to the self-face were shorter than RTs to fearful and happy faces. This is in line with numerous studies showing that detection of one’s own face is much faster than detection of other faces (for review see: Bortolon and Raffard, 2018).

The main limitation of our study is the lack of an additional control condition that presents a mixture of the extreme familiarity and emotional load factors, as it is the case for the self, e.g. a best friend’s or partner’s face. Inclusion of such faces would enable us to test whether effects similar to those observed for the self-face can be observed for faces that are not only as familiar as the self-face but also subjectively very significant. Such an approach would reveal whether the differences between the self-face and other (emotional and neutral) faces were self-specific only or whether other highly familiar and highly significant faces were processed similar to the self-face. Future studies that expand the current paradigm by inclusion of such an additional condition may contribute to the discussion on the issue of whether the self is a higher-order function or a fundamental function of the brain (Northoff, 2016) and may provide some additional arguments in favour of one of the opposite views.

In conclusion, our ERP results as well as the results of RSA and cluster-based permutation tests consistently showed differences between the self-face and other (emotionally negative, emotionally positive and emotionally neutral) faces. These findings strongly suggest that self-face processing does not resemble the processing of emotional faces, thus implying that self-referential processing is truly reflective of self. They also seem to point to the crucial role of subjective significance as a leading factor in the prioritized self-face processing. Direct comparisons of the self-face *vs* emotional faces processing may be applied in the further experimental pursuit of the mechanisms underlying self-referential processing and may shed new light on the operations that are necessary for self-awareness. In this context, the winner-takes-all characteristic of the self-preference effects and its temporal resolution seems to be particularly relevant.

## Supplementary Material

nsab020_Supp
