# Supplementary material for: Self-face and emotional faces—are they alike?
Source: Soc Cogn Affect Neurosci. 2021 Feb 8;16(6):593–607. doi: 10.1093/scan/nsab020 (PMC8218856; doi:10.1093/scan/nsab020)
Supplement: nsab020_Supp [file nsab020_supp.zip › scan-20-279-File007.docx]

**Supplementary Material**

**Figure S1.** Grand-average ERPs at all 62 electrodes


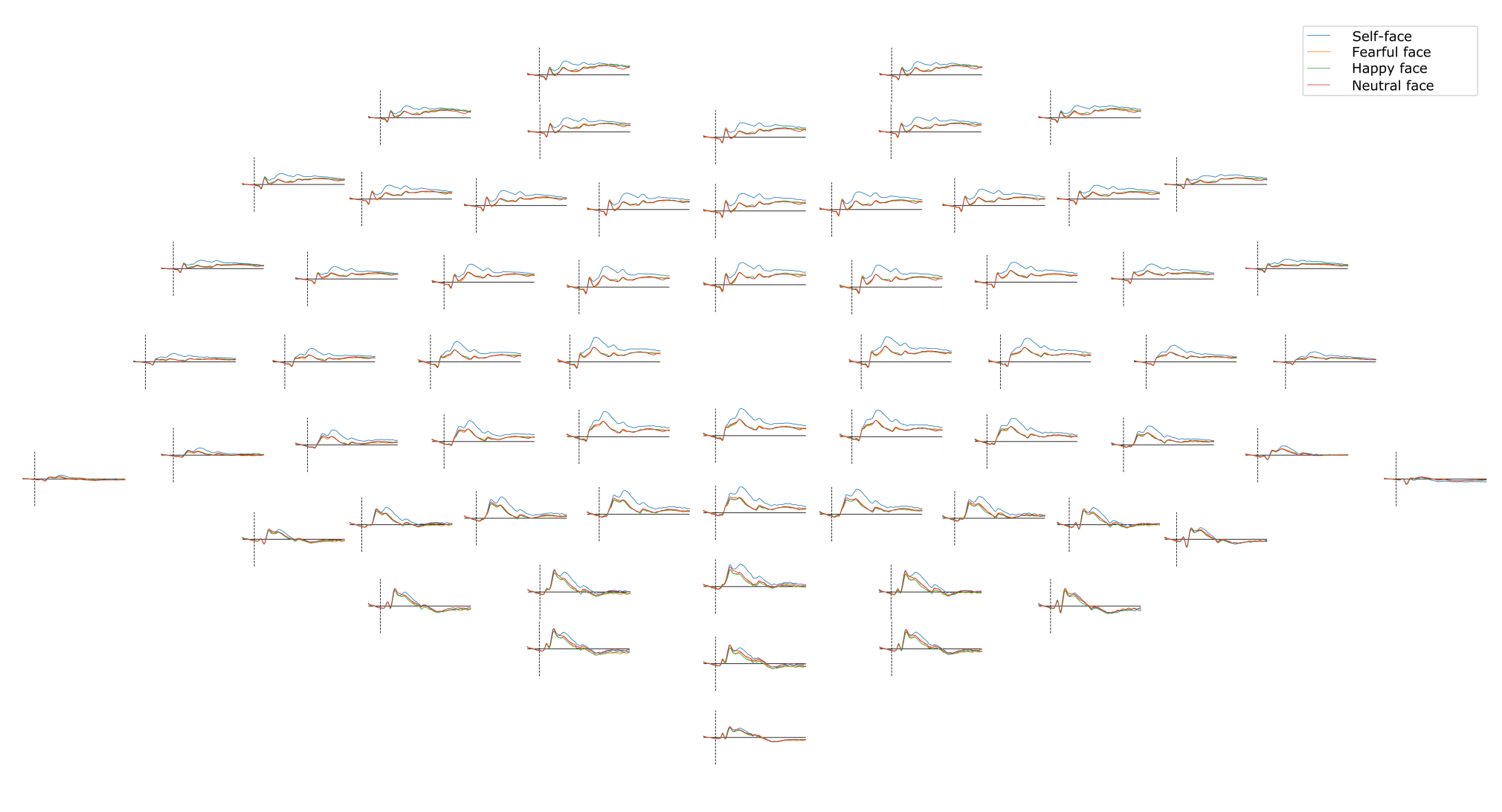


**Figure S2.** Results of permutation tests for 32 electrodes that were not presented in Fig. 2.

**
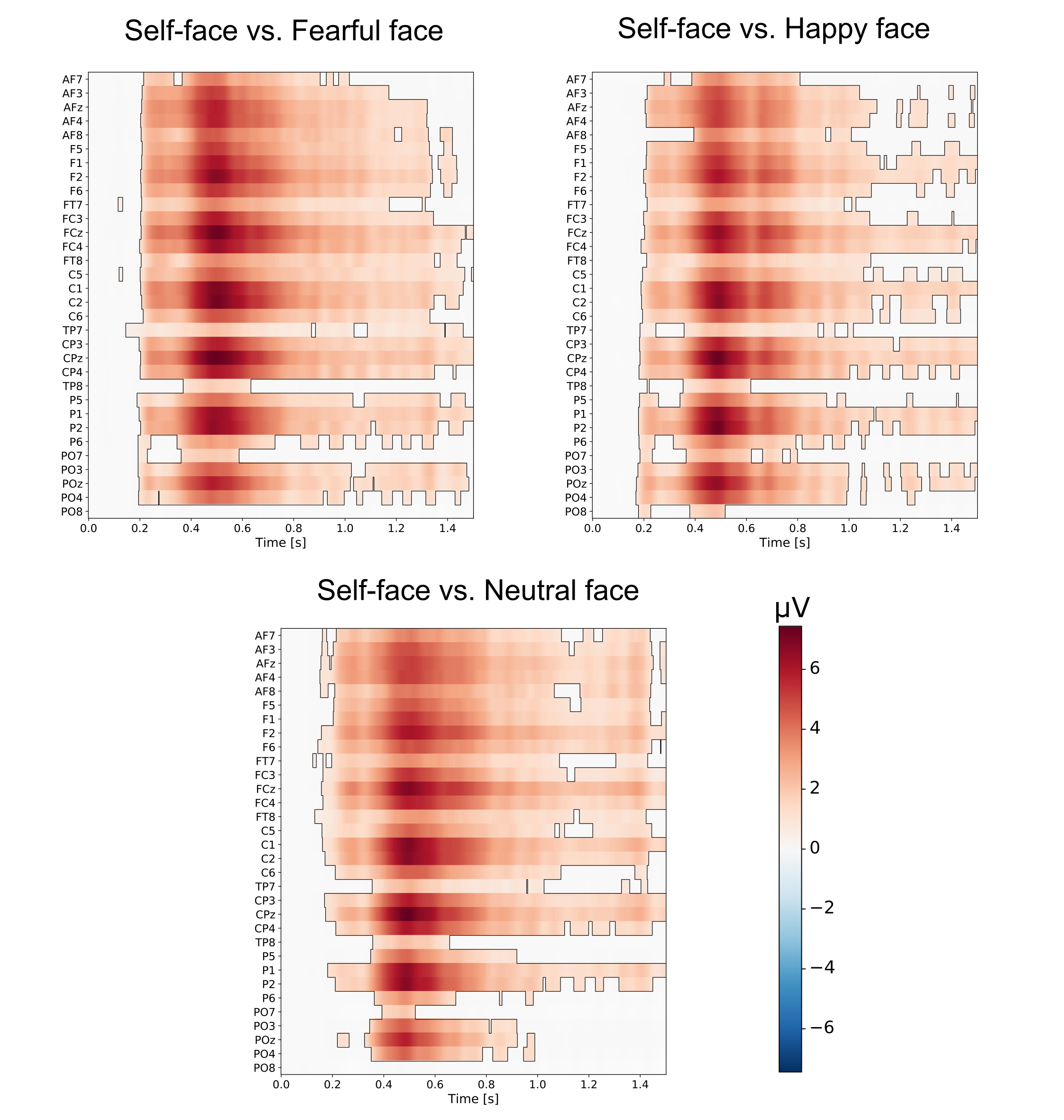
**

**Figure S3.** Grand-average ERPs associated with processing of happy, fearful, and neutral faces in the N170 time-window for pooled P6, P8, and PO8 electrodes.


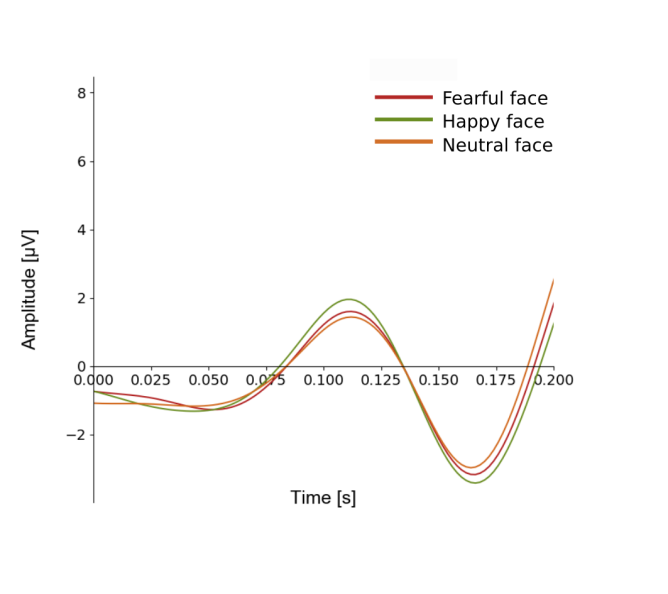


The N170 is related to early stages of face encoding (Eimer, 2000). Some studies have showed that N170 is influenced by emotional expressions displayed by facial stimuli (e.g. Blau et al., 2007). In the present study, the amplitude of this ERP component was measured and analyzed as a peak-to-peak against the preceding P100 (Cygan et al. 2004). Planned comparisons revealed that N170 associated with the processing of emotional faces significantly differed from N170 to neutral faces (happy vs. neutral: t(28) = -2.772, *P* = .006; fearful vs. face: t(28) = -2.437, *P* = .011)

Blau, V. C., Maurer, U., Tottenham, N., & McCandliss, B. D. (2007) The face-specific N170 component is modulated by emotional facial expression. Behavioral Brain Function, 3, 7. doi:10.1186/1744-9081-3-7

Cygan, H. B., Tacikowski, P., Ostaszewski, P., Chojnicka, I., & Nowicka, A. (2014) Neural correlates of own name and own face detection in autism spectrum disorder. PLoS One, 9 (1), e86020. https://doi.org/10.1371/journal.pone.0086020

Eimer, M. (2000) Event-related brain potentials distinguish processing stages involved in face perception and recognition. Clinical Neurophysiology 111: 694–705. doi: 10.1016 /S1388-2457(99)00285-0

**Figure S4.** LDA decoding of self-, happy, fearful, and neutral faces (colored bars indicate significant effects).


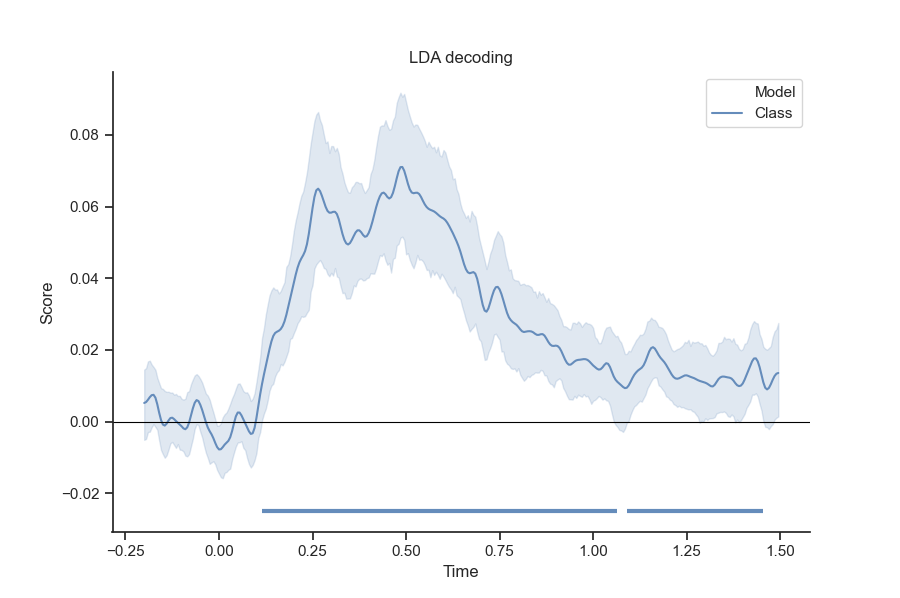


To assess whether other experimental conditions (neutral, happy and fearful expressions) were differentiated and to investigate the possible time dynamics of these effects, we used a

linear discriminant analysis (LDA). LDA is a statistical method used to find a linear combination of features (in our case amplitude values registered at each sensor) that optimally separates two or more classes of objects (i.e. experimental conditions). In neuroscience, the results of such an analysis are often interpreted as the amount of information about a specific category affiliation that is encoded in the system. This is related to the fact that the operations necessary for a linear readout (weighted sum and threshold operations) can be implemented in single unit in a biologically plausible fashion and indicate the presence of explicitly accessible information (e.g. Hung et al., 2005).

Hung, C. P., Kreiman, G., Poggio, T., & DiCarlo, J. J. (2005). Fast readout of object identity from macaque inferior temporal cortex. Science, 310(5749), 863-866.

**Figure S5.** LDA decoding of happy, fearful, and neutral faces (colored bars indicate significant effects).

**
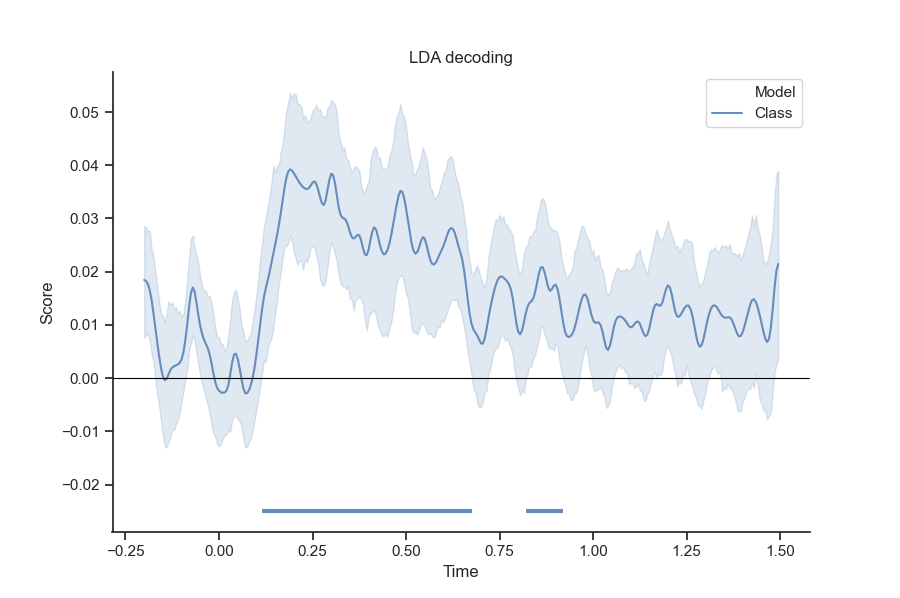
**
